# Effects of allopurinol and febuxostat on uric acid transport and transporter expression in human umbilical vein endothelial cells

**DOI:** 10.1371/journal.pone.0305906

**Published:** 2024-06-21

**Authors:** Karel H. van der Pol, Jan Koenderink, Jeroen J. M. W. van den Heuvel, Petra van den Broek, Janny Peters, Imke D. W. van Bunningen, Jeanne Pertijs, Frans G. M. Russel, Jim Koldenhof, Wim J. Morshuis, Joris van Drongelen, Tom J. J. Schirris, Andries van der Meer, Gerard A. Rongen

**Affiliations:** 1 Department of Internal Medicine, Radboud University Medical Center, Nijmegen, The Netherlands; 2 Department of Pharmacology and Toxicology, Radboud University Medical Center, Nijmegen, The Netherlands; 3 Applied Stem Cell Technologies, University of Twente, Enschede, The Netherlands; 4 Department of Cardio-thoracic Surgery, Radboud University Medical Center, Nijmegen, The Netherlands; 5 Department of Obstetrics and Gynaecology, Radboud University Medical Center, Nijmegen, The Netherlands; Helwan University, EGYPT

## Abstract

Uric acid induces radical oxygen species formation, endothelial inflammation, and endothelial dysfunction which contributes to the progression of atherosclerosis. Febuxostat inhibits BCRP- and allopurinol stimulates MRP4-mediated uric acid efflux in human embryonic kidney cells. We hypothesized that endothelial cells express uric acid transporters that regulate intracellular uric acid concentration and that modulation of these transporters by febuxostat and allopurinol contributes to their different impact on cardiovascular mortality. The aim of this study was to explore a potential difference between the effect of febuxostat and allopurinol on uric acid uptake by human umbilical vein endothelial cells. Febuxostat increased intracellular uric acid concentrations compared with control. In contrast, allopurinol did not affect intracellular uric acid concentration. In line with this observation, febuxostat increased mRNA expression of *GLUT9* and reduced *MRP4* expression, while allopurinol did not affect mRNA expression of these uric acid transporters. These findings provide a possible pathophysiological pathway which could explain the higher cardiovascular mortality for febuxostat compared to allopurinol but should be explored further.

## 1. Introduction

Uric acid is the final product of purine metabolism in humans. The enzyme xanthine oxidase converts hypoxanthine and xanthine to uric acid. Uric acid is subsequently excreted by the kidneys and gastrointestinal tract [1]. Multiple transporters are involved in excretion of uric acid in the kidney [1, 2], and the gastrointestinal tract [3]. Uric acid transporters are also expressed on vascular endothelial cells, such as human umbilical vein endothelial cells (HUVEC), but reports differ on what transporters are expressed [4, 5]. The presence of uric acid transporters on human aortic endothelial cells (HAEC) is unknown.

Hyperuricemia is associated with hypertension [6, 7], chronic kidney disease [8], type two diabetes mellitus [9], and atherosclerotic cardiovascular disease [10, 11]. Whether uric acid plays a causal role in these diseases, or is merely a risk marker, has been subject of debate. Preclinical evidence indicates the observed increase in cardiovascular risk is mediated by effects of uric acid and xanthine oxidase activity on the vascular endothelium via multiple pathways. These include effects on radical oxygen species (ROS) formation, endothelial inflammation, and endothelial dysfunction, ultimately contributing to the progression of atherosclerosis [12]. Uric acid transporters located on vascular endothelial cells also play a role in intracellular uric acid accumulation, ROS formation, and endothelial inflammation [4, 13, 14]. Meta-analysis have found that xanthine oxidase inhibitors (XOI) can increase flow-mediated dilatation [15, 16], reduce blood pressure [17], and decrease risk of major adverse cardiovascular events [18, 19]. However, high quality randomized controlled trial data on the effects of XOI compared with no treatment or placebo on major adverse cardiovascular events in patients with hyperuricemia is lacking.

Serum uric acid can be lowered with XOI such as allopurinol and febuxostat, and uric acid transporter inhibitors like benzbromarone. While XOI reduce uric acid production by inhibiting xanthine oxidase, uric acid transport inhibitors increase excretion of uric acid in the kidney [1]. In the CARES trial, a randomized controlled trial comparing the cardiovascular safety of allopurinol and febuxostat, an increased risk of all-cause and cardiovascular mortality was observed in the febuxostat-treated group [20]. The subsequent FAST trial did not find significant differences between allopurinol and febuxostat [21]. It should be noted that important differences in baseline prevalence of atherosclerotic disease exist, 100% versus 33% for CARES and FAST respectively. This indicates that the CARES population was more vulnerable to cardiovascular events than the FAST population. Earlier studies found that allopurinol can stimulate multidrug resistance protein 4 (MRP4)-mediated uric acid efflux [22], and febuxostat can inhibit breast cancer resistance protein (BCRP)-mediated uric acid efflux in human embryonic kidney cells [23].

Based on the above-mentioned data we hypothesized that allopurinol and febuxostat may affect uric acid transporters as off-target effect, and consequently intracellular uric acid concentration, in vascular endothelial cells. Possible differences in intracellular uric acid concentration may affect endothelial inflammation and ultimately cardiovascular mortality. The goal of the current study was to explore a potential difference between the effect of febuxostat and allopurinol on uric acid uptake by human umbilical vein endothelial cells.

## 2. Methods

### 2.1 Materials

Human umbilical cords were collected anonymously from the obstetrics department in our hospital. Aortic tissue was collected anonymously from the department of thoracic surgery from patients who had undergone aortic surgery in our hospital. Collagenase type 2 (#LS004176) was obtained from Worthington Biochem. M199 medium (#22340020), RPMI 1640 medium (#22409015), newborn calf serum (#26010074), trypsin (#25300054), L-glutamine (#25030081), penicillin/streptomycin (#15140122), GlutaMAX (#35050038), and pyruvate (#11360039) was obtained from Gibco. Human heat inactivated serum (#ISERABOTC100ML) was obtained from Innovative Research. Heparin was obtained from the apothecary in our hospital. Calf brain derived growth factors were obtained from the department of pediatrics in our hospital. Gelatin (#48720), ammonium formate (#70221), formic acid (#33015), and uric acid (#U2625) was obtained from Sigma-Aldrich. Epidermal Growth Factor (EGF) (#C-60170), Recombinant Human Fibroblast Growth Factor 5 (FGF-5) (#C-60350), Recombinant Human Long R3 Insulin Like Growth Factor 1 (IGF-1) (#C-60839), and Recombinant Human Vascular Endothelial Cell Growth Factor 165 (VEGF-165) (#C-64420) was obtained from PromoCell. Qiagen RNeasy mini kit (#74104) was obtained from Qiagen. Hoechst 33342 (#H3570), M-MLV Transcriptase (#28025013), TaqMan Universal PCR Master Mix (#4304437), and gene-specific primer-probe sets: glyceraldehyde-3-phosphate dehydrogenase (GAPDH), Hs99999905_m1; glucose transporter 9 (GLUT9), Hs00417125_m1; urate transporter 1 (URAT1), Hs00375985_m1; organic anion transporter 1 (OAT1), Hs00537914_m1; organic anion transporter 3 (OAT3), Hs00188599_m1; organic anion transporter 4 (OAT4), Hs00945829_m1; BCRP, Hs00184979_m1; sodium-dependent dicarboxylate transporter (NaDC3), Hs00955744_m1; MRP4, Hs00195260_m1 were obtained from Life Technologies Invitrogen. ^13^C_3_ uric acid (#TRC-U829204) was obtained from Toronto Research Chemicals. Bio-Rad protein assay (#500–0006) was obtained from Bio-Rad. All other materials were obtained from the central laboratory dispensary in our hospital.

### 2.2 HUVEC isolation and culturing

Human umbilical cords were stored in phosphate buffered saline (PBS) at 4°C until processing. To isolate HUVEC a cannula was placed in either end of the umbilical vein and tied down with a surgical suture. A syringe was placed on the cannula, the cord was flushed several times with PBS to remove blood from the umbilical vein and subsequently incubated with 0.1% (w/v) collagenase type 2 in M199 medium at 37°C for 10 minutes. After gently massaging the cord, it was washed with M199 medium to harvest the HUVEC. The collection tube was centrifuged for 5 minutes at 300x g. The cell pellet was resuspended in isolation medium (M199 HEPES containing 5.5mM D-glucose, to which human heat inactivated serum, newborn calf serum, L-glutamine, heparin, penicillin/streptomycin, and calf brain derived growth factors was added). Cells were then seeded to a culture flask coated with 0.1% (w/v) Gelatin in Milli-Q and placed in an CO_2_ incubator (37°C, 5% CO_2,_ 95% ambient air). After 24 hours the flask was washed 2–3 times with PBS to remove all remaining erythrocytes. Culture medium (RPMI 1640 Dutch modification containing 11.11 mM D-glucose, to which human heat inactivated serum, newborn calf serum, GlutaMAX, pyruvate, heparin, EGF, FGF-5, IGF-1, and VEGF-165 was added) was refreshed every 2–3 days and cells were passaged when reaching confluency. At this point cells could optionally be frozen. HUVEC were stored at -80°C for at least 4 hours in cell freezing containers ensuring a controlled cooling rate of -1°C/minute after which cells were transferred to -196°C until used for assays. For all further experiments HUVEC no higher than passage 5 were used.

### 2.3 HAEC isolation

Aortic tissue was placed in PBS on ice until processing, which was done within one hour of tissue retrieval. The tissue was rinsed with PBS to remove blood. It was immersed in PBS containing 0.06% collagenase in a dish and incubated at 37°C in a moist incubator. Only the luminal side was exposed to collagenase. After 30 minutes, the collagenase was diluted in 1:2 mixture of medium and this was collected in a tube. The collagenase exposed side of the aortic tissue was rinsed with medium. This was also collected in a tube. Both tubes were centrifuged for 5 minutes at 300x g at 4°C. The supernatant was aspirated and the pellets were washed twice with 1 mL of ice-cold PBS. The supernatant was then aspirated. After this step, the tubes were stored at -80°C or immediately used for mRNA isolation.

### 2.4 Uric acid transport and protein concentration

HUVECs were seeded to 24-wells plates coated with 1% w/v gelatin at a density of 20.000 cells/cm^2^. Maturation medium (RPMI 1640 medium with human heat inactivated serum, newborn calf serum, GlutaMAX and pyruvate) was used. Cells were allowed to reach confluency and mature for 1 week, and maturation medium was replaced every 2–3 days. At day 7 medium was aspirated and cells were washed with PBS. Subsequently, cells were incubated with 1ml assay medium (RPMI 1640 serum free medium with GlutaMAX and pyruvate) in combination with uric acid (100μM and 350μM) and allopurinol (20μM and 40μM), febuxostat (10μM and 20μM), or their solvents as control (1mM sodium hydroxide (NaOH) for allopurinol and 0.1%(v/v) dimethyl sulfoxide (DMSO) for febuxostat). Uric acid was directly dissolved in culture medium. Uric acid concentrations higher than 350μM were not used as this resulted in crystal formation. Allopurinol and febuxostat concentrations used in these experiments were based on reported C_max_ concentrations of clinically relevant doses in humans [24, 25]. Twenty-four hours later, cells were washed twice with ice-cold 400μl Hanks’ Balanced Salt Solution (HBSS) + 0.5% bovine serum albumin (BSA), and then once with ice-cold HBSS without BSA. The cells were then lysed with 100μl 50% methanol. Subsequently, 100μl 0.1μg/ml internal standard (^13^C_3_ uric acid) was added and mixed well. Thereafter, samples were centrifuged and supernatant was collected. Uric acid concentrations in the supernatant were quantified by LC-MS/MS using an Acquity UPLC (Waters, Milford, MA, USA) coupled to a Xevo TQ-S (Waters) triple quadropole mass spectrometer. The compounds were separated on an Acquity UPLC HSS T3 column (Waters, 2,1 x 100mm, 1,8μm). The mobile phase consisted of solvent A (1mM Ammonium formate, 0.1%(v/v) formic acid in water) and solvent B (0.1%(v/v) formic acid methanol). The mobile phase gradient was: initial, 0% B; 2 min, 50% B; 2–4 min, 100% B; 4-5min, 0% B. The column temperature was set at 40°C, and the flow rate was 300μl/min. The effluent from the UPLC was passed directly into the electrospray ion source. Negative electrospray ionization was achieved using nitrogen as a desolvation gas with ionization voltage at 0.5 kVolt. The source desolvation temperature was set at 500°C and argon was used as collision gas. Negative mode with multiple reaction monitoring (MRM) was used for the quantitative analysis of uric acid and ^13^C_3_ -uric acid. The following MRM-transitions were used: *m/z* 166.8 (parent ion) to *m/z* 123.9 and 96.0 (both product ions) for uric acid and m/z 169.8 (parent ion) to m/z 126.7 and 98.1 (both product ions) for ^13^C_3_-uric acid. To determine protein concentrations, the above-mentioned steps up to lysing of the cells were followed, but instead, cells were lysed with 200 μl KOH. Protein content was then determined using the Bio-Rad protein assay. Six individual donors were used for these measurements.

### 2.5 Transporter expression in HUVEC and HAEC

Quantitative PCR (qPCR) was used to determine the expression of genes involved in uric acid transport. To harvest HUVEC, culture medium was aspirated, and cells were washed with PBS. Trypsin was then added to each well and after a few minutes cells detached from the surface. Culture medium was then added to deactivate trypsin. The cell suspension was transferred to a tube, centrifuged, and washed three times with PBS. The cell pellet could then be frozen or used directly for the next step. mRNA was isolated using the Qiagen RNeasy mini kit. cDNA was prepared using M-MLV Transcriptase, with a sample containing water instead of mRNA as control. The mRNA expression levels were determined using gene-specific primer-probe sets for the following uric acid transporter genes: *GLUT9*, *URAT1*, *OAT1*, *OAT3*, *OAT4*, *BCRP*, *NaDC3*, and *MRP4*. *GAPDH* was used as housekeeping gene. The HUGO gene nomenclature committee approved gene name, gene symbol, and the alias used in this article are summarized in Table 1 [26, 27].

**Table 1:** Overview of official gene symbol, gene name, and alias used in the text.

Gene Symbol^a^	Gene Name^a^	Alias^b^
*GAPDH*	glyceraldehyde-3-phosphate dehydrogenase	-
*ABCC4*	ATP binding cassette subfamily C member 4	*MRP4*
*ABCG2*	ATP binding cassette subfamily G member 2	*BCRP*
*SLC2A9*	solute carrier family 2 member 9	*GLUT9*
*SLC13A3*	solute carrier family 13 member 3	*NaDC3*
*SLC22A6*	solute carrier family 22 member 6	*OAT1*
*SLC22A8*	solute carrier family 22 member 8	*OAT3*
*SLC22A11*	solute carrier family 22 member 11	*OAT4*
*SLC22A12*	solute carrier family 22 member 12	*URAT1*

^a^ as recommended by the HUGO Gene Nomenclature Committee

^b^ alias commonly used in literature

The qPCR was performed with the TaqMan Universal PCR Master Mix, using a sample containing water instead of cDNA, as control. This was analyzed using Bio-Rad CFX Manager. The results were portrayed as Ct values and normalized to *GAPDH*. Differences between groups were calculated by the Livak (delta-delta Ct) method. Six individual donors were used for mRNA expression of *GLUT9* and *NaDC3*, and four individual donors were used for mRNA expression of the other uric acid transporters in HUVEC. Three individual donors were used for mRNA expression of *OAT4*, *NaDC3*, *GLUT9*, *MRP4*, *BCRP*, and *URAT1* in HAEC; one donor was used for mRNA expression of *OAT1* and *OAT3* in HAEC.

After the initial determination of expression of uric acid transporter genes, the effects of uric acid, allopurinol and febuxostat on the expression of *GLUT9*, *BCRP*, and *MRP4* relative to *GAPDH* was studied in HUVEC. For this assay cultured HUVEC were incubated for 24 hours with uric acid (0μM, 100μM, and 350μM) in combination with allopurinol (40μM) or febuxostat (20μM). HUVEC not exposed to uric acid nor allopurinol/febuxostat were used as control. mRNA expression was then determined using the method described above. Four individual donors were used for these measurements.

### 2.6 Cell count

Cell count was performed using a previously described protocol [28]. Briefly, HUVECs were seeded on a 96-wells plate coated with 1% w/v gelatin at a density of 20.000 cells/cm^2^ and were allowed to reach confluency and mature for 1 week. HUVEC were then incubated with assay medium in combination with uric acid (100μM and 350μM) and allopurinol (20μM and 40μM), febuxostat (10μM and 20μM), or their solvents as control (NaOH for allopurinol and DMSO for febuxostat). Three replicates of each concentration were tested. After 24-hour exposure, nuclei were stained using Hoechst 33342 (20μg/mL) for 20 min at 37°C. Fluorescence was imaged on a BD Pathway 855 high-throughput microscope (Becton Dickinson (BD) Bioscience). The number of nuclei per image was analyzed using a previously used CellProfiler protocol [29]. Four individual donors were used for these measurements.

### 2.7 Ethics statement

For this study ethical approval was obtained from the research ethics committee of the Radboud University Nijmegen Medical Centre (approval number: 2019–5413). The need for obtaining informed consent was waived by the committee as all donor tissue was collected anonymously. Donor tissue was collected between the 27^th^ of May 2019 and 28^th^ of June 2021.

### 2.8 Statistical analyses

Data comparing mRNA expression of uric acid transporters in HUVEC and HAEC is presented as mean ± SD. Independent t-tests were used to compare uric acid transporter expression between HUVEC and HAEC. All other data (i.e data from experiments studying the effects of allopurinol and febuxostat) is reported as median (range) and was normalized to the matched control condition. Uric acid transport data was corrected for the protein concentration in each respective condition. Friedman’s test was used for three or more groups, and Wilcoxon signed-rank test was used for 2 groups. When the Friedman’s test yielded a significant result, Dunn’s multiple comparisons test was applied as follow-up test. Each condition was compared against all other conditions within their group (i.e. all conditions shown in the same figure panel). Nonparametric tests were used, as data was normalized to their respective matched control and therefore variances were not equal between groups. P-values <0.05 were considered statistically significant. Data was analyzed using Graphpad Prism 9.0.0.

## 3. Results

### 3.1 Influence of allopurinol and febuxostat on intracellular uric acid concentrations in HUVEC

**Fig 1:**
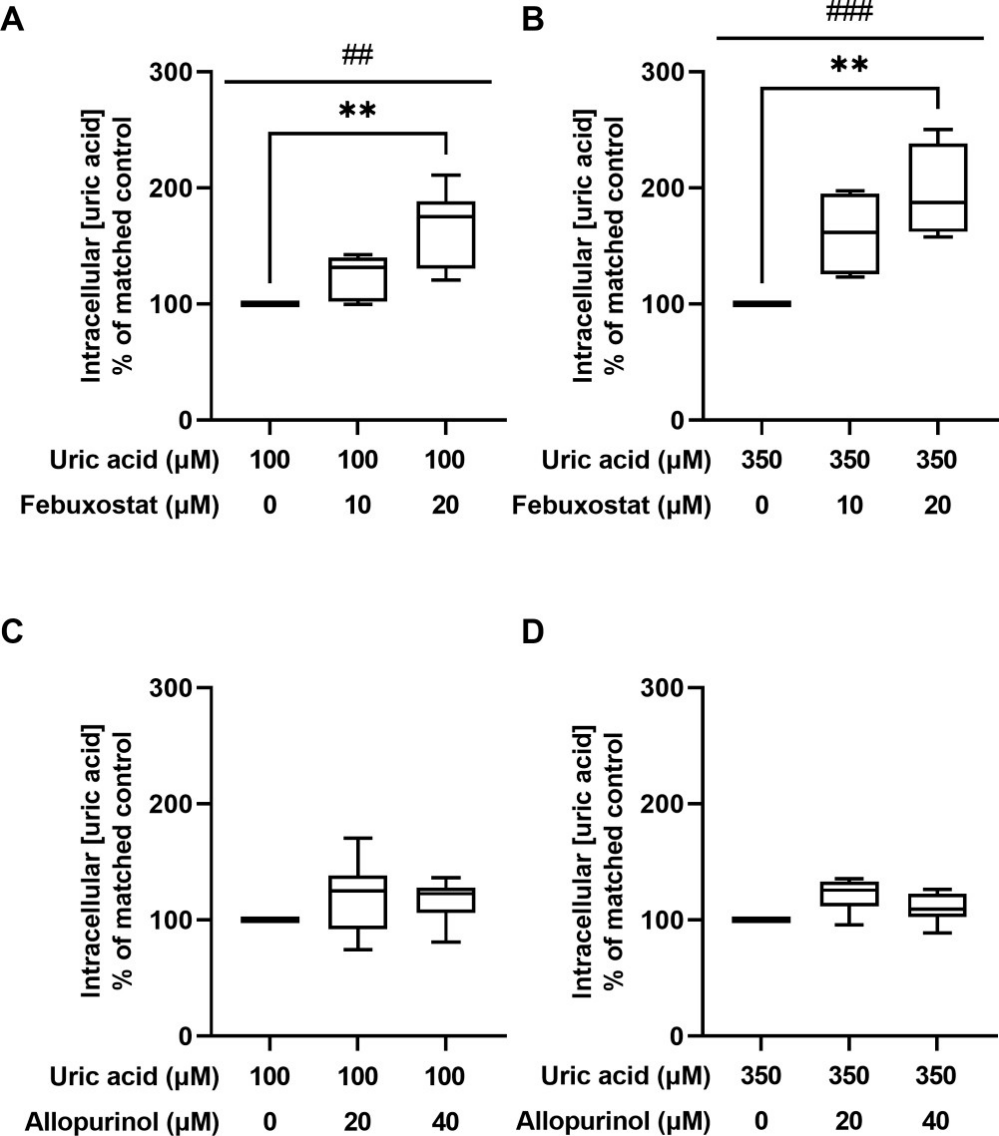
Intracellular uric acid concentrations in HUVEC treated with allopurinol and febuxostat compared with matched control condition. (**A**) effect of febuxostat in combination with 100μM uric acid on intracellular uric acid concentrations; (**B**) effect of febuxostat in combination with 350μM uric acid on intracellular uric acid concentrations; (**C**) effect of allopurinol in combination with 100μM uric acid on intracellular uric acid concentrations; (**D**) effect of allopurinol in combination with 350μM uric acid on intracellular uric acid concentrations; Hashtags represent P-values calculated with Friedman’s test, ##P<0.01, ###P<0.001; Asterixis represent P-values calculated with Dunn’s test, **P<0.01; Box represents median, 25^th^, and 75^th^ percentile, whiskers represent range.

Medium containing 350μM uric acid significantly increased intracellular uric acid concentration by 258% (167–383%) compared with medium containing 100μM uric acid (S1 Fig). Febuxostat 10μM and 20μM, dissolved in medium containing 100μM uric acid, increased intracellular uric acid concentrations by 32% (0–42%) and 75% (21–111%) compared with control (Friedman’s P<0.01) (Fig 1A). Dunn’s test showed a significant difference for control versus febuxostat 20μM (P<0.01), but not for control versus febuxostat 10μM, or febuxostat 10μM versus 20μM. Febuxostat 10μM and 20μM, dissolved in medium containing 350μM uric acid, increased intracellular uric acid concentrations by 62% (23–98%) and 88% (58–150%) compared with control (Friedman’s P<0.001) (Fig 1B). Dunn’s test showed a significant difference for control versus febuxostat 20μM (P<0.01), but not for control versus febuxostat 10μM, or febuxostat 10μM versus 20μM. Allopurinol 20μM and 40μM did not significantly affect intracellular uric acid concentrations neither dissolved in medium containing 100 μM nor 350μM uric acid (Fig 1C and 1D).

### 3.2 mRNA expression of uric acid transporters in HAEC and HUVEC

**Fig 2:**
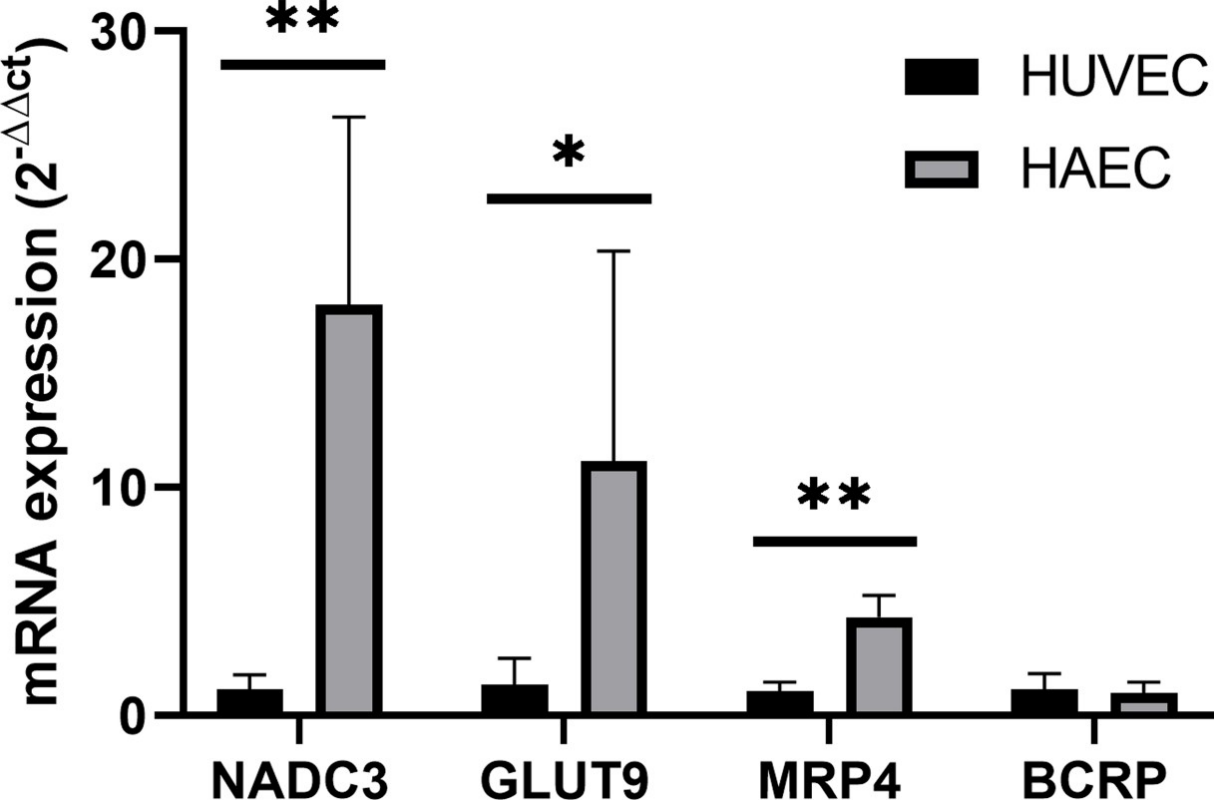
Comparison of mRNA expression of uric acid transporters in HUVEC and HAEC calculated with the Livak method. *P<0.05, **P<0.01; Each bar represent mean ±SD.

*BCRP*, *MRP4*, and *GLUT9* were expressed in HUVEC and HAEC at mRNA level. *NaDC3*, encoding a carboxylate transporter associated with serum uric acid, was also expressed. *OAT1*, *OAT3*, *OAT4*, and *URAT1* were not detected. Expression of *GLUT9*, *MRP4*, and *NaDC3* was significantly higher in HAEC compared to HUVEC (Fig 2).

### 3.3 Influence of allopurinol and febuxostat on mRNA expression of uric acid transporters in HUVEC

**Fig 3:**
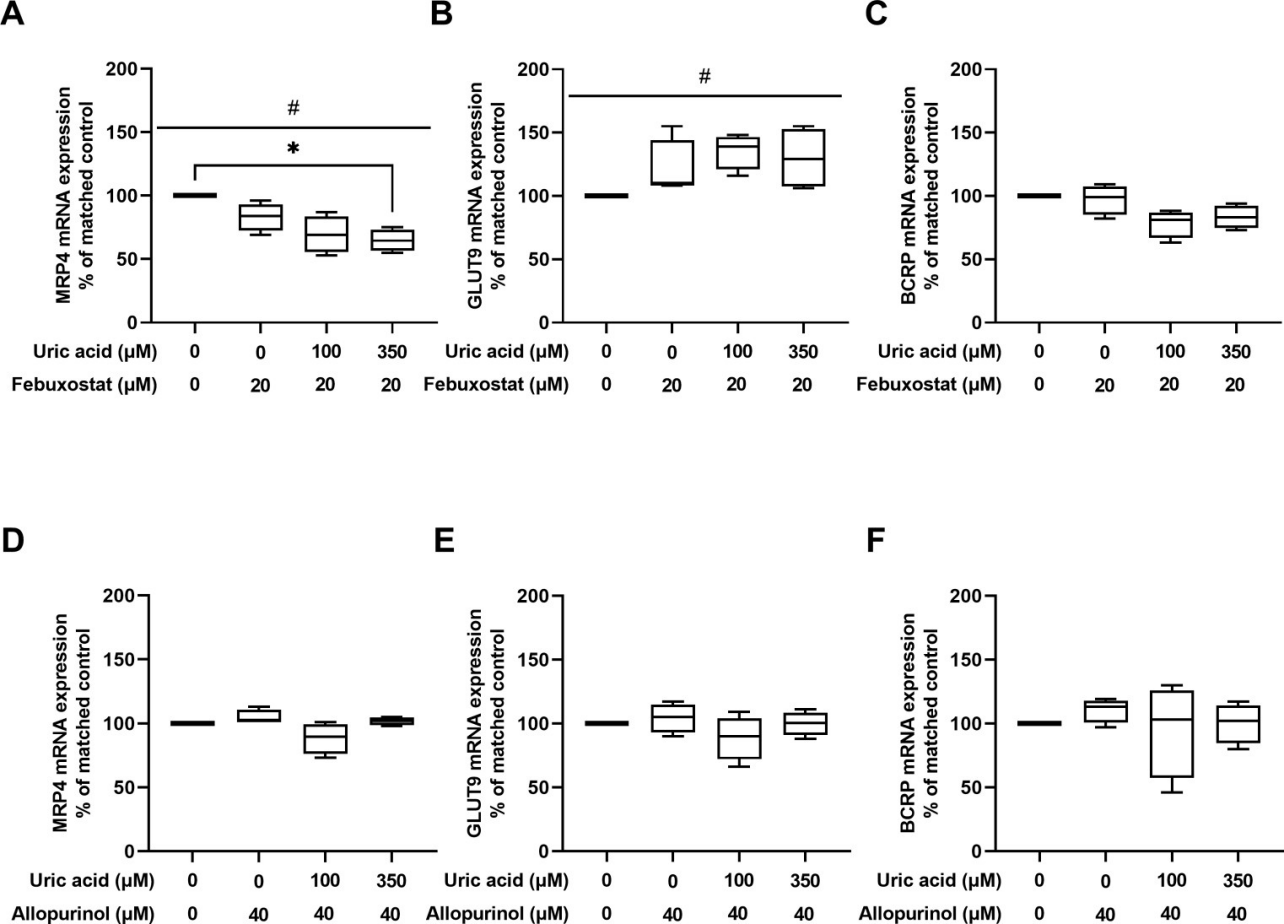
mRNA expression of uric acid transporters in HUVEC treated with allopurinol and febuxostat compared with matched control condition. (**A**) effect of febuxostat in combination with uric acid on MRP4 mRNA expression; (**B**) effect of febuxostat in combination with uric acid on GLUT9 mRNA expression; (**C**) effect of febuxostat in combination with uric acid on BCRP mRNA expression; (**D**) effect of allopurinol in combination with uric acid on MRP4 mRNA expression; (**E**) effect of allopurinol in combination with uric acid on GLUT9 mRNA expression; (**F**) effect of allopurinol in combination with uric acid on BCRP mRNA expression; Hashtags represent P-values calculated with Friedman’s test, #P<0.05; Asterixis represent P-values calculated with Dunn’s test, *P<0.05; Box represents median, 25^th^, and 75^th^ percentile, whiskers represent range.

Febuxostat 20μM, dissolved in medium containing 0μM, 100μM, or 350μM uric acid, decreased *MRP4* mRNA expression by 16% (4–31%), 31% (13–47%), and 36% (25–45%) compared with control (Friedman’s P<0.05) (Fig 3A). Dunn’s test showed a significant difference for control versus febuxostat 20μM with uric acid 350μM (P<0.05), but not for all other comparisons.

Febuxostat 20μM, dissolved in medium containing 0μM, 100μM, or 350μM uric acid, increased *GLUT9* mRNA expression by 10% (8–55%), 39% (16–48%), and 29% (6–55%) compared with control (Friedman’s P<0.05) (Fig 3B). Dunn’s test did not show a significant difference for any of the comparisons.

Febuxostat 20μM, dissolved in medium containing 0μM, 100μM, or 350μM uric acid, did not significantly affect *BCRP* mRNA compared with control (Fig 3C).

Allopurinol 40μM, dissolved in medium containing 0μM, 100μM, or 350μM uric acid, did not significantly affect *GLUT9*, *MRP4*, nor *BCRP* mRNA expression compared with control (Fig 3D–3F).

### 3.4 Influence of allopurinol and febuxostat on cell count in HUVEC

**Fig 4:**
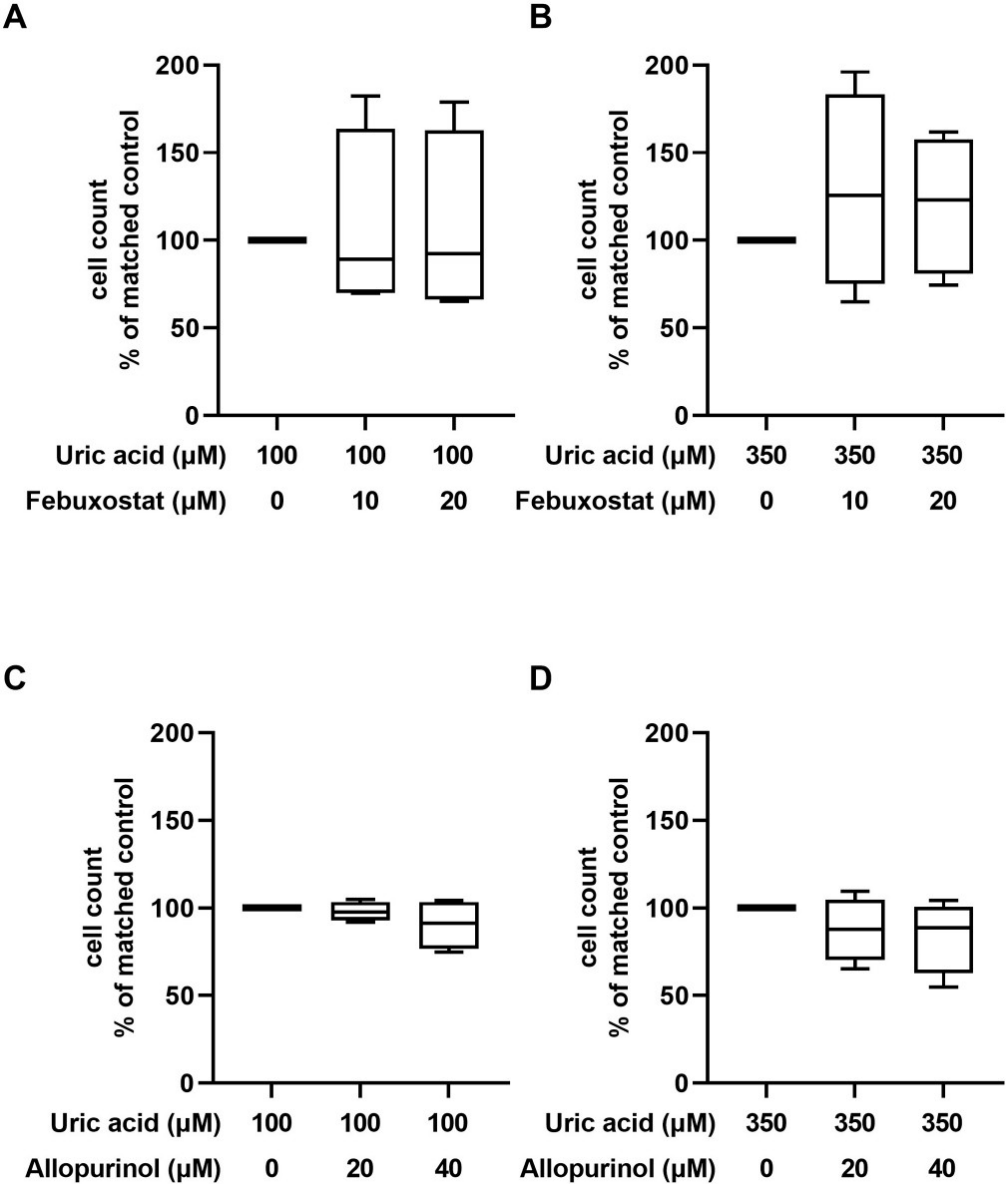
Cell count in HUVEC treated with allopurinol and febuxostat compared with matched control condition. **(A)** effect of febuxostat in combination with 100μM uric acid on cell count; (**B**) effect of febuxostat in combination with 350μM uric acid on cell count; (**C**) effect of allopurinol in combination with 100μM uric acid on cell count; (**D**) effect of allopurinol in combination with 350μM uric acid on cell count; Box represents median, 25^th^, and 75^th^ percentile, whiskers represent range.

There was no significant difference in cell count between medium containing 100μM and 350μM uric acid (S2 Fig). Neither febuxostat nor allopurinol, dissolved in medium containing 100μM or 350μM uric acid, significantly affected cell count compared with control (Fig 4A–4D).

## 4. Discussion

To our knowledge, this is the first report on the effect of allopurinol and febuxostat on intracellular uric acid concentration in HUVEC and the expression of uric acid transporters in HAEC. Intracellular uric acid concentration has previously been shown to affect endothelial inflammation and oxidative stress [4, 13, 14]. This contributes to the progression of atherosclerosis and subsequent cardiovascular disease and mortality [12]. The current results provide a possible pathophysiological pathway explaining the increased cardiovascular mortality for febuxostat compared with allopurinol [20].

In our study we used LC-MS/MS to measure intracellular uric acid concentrations which is a reliable and accurate method of measuring uric acid in the intracellular environment [30].

Our study also has several limitations. Firstly, while there were no qualitative differences in uric acid transporter expression between HUVEC and HAEC, we did not test whether the quantitative differences in mRNA expression of uric acid transporters translated into differences at the protein level or in intracellular uric acid concentration. Differences in the expression of uric acid transporters may influence the accumulation of intracellular uric acid, consequently impacting inflammation in these endothelial cells. Furthermore, increased expression of uric acid transporters might influence the susceptibility of cells to the modulation of uric acid uptake by febuxostat. We were unable to perform these experiments due to limited aortic donor material.

Secondly, while mRNA expression of uric acid transporter was significantly affected by febuxostat in HUVEC, we did not measure protein expression of these transporters. Therefore, it is unknown whether uric acid transporter expression was affected at the protein level.

Thirdly, our data do not permit us to differentiate between a direct drug effect or an effect of intracellular uric acid on uric acid transporter expression. A previous study showed that intracellular uric acid accumulation, independent of any drug, resulted in increased intracellular ROS content and decreased BCRP expression at the cell membrane, while MRP4 expression was not affected [13]. Concomitant incubation with N-acetylcysteine, an anti-oxidant, blocked the uric acid-mediated ROS increase and abolished the effect on BCRP expression. Uric acid decreased Akt phosphorylation which was reversed by N-acetylcysteine and incubation with a phosphoinositide 3-kinase (PI3K)/Akt pathway inhibitor reduced BCRP. These results indicate that uric acid affects BCRP expression at the cell membrane via induction of oxidative stress and decreased phosphorylation of Akt. Of note, no effect on *GLUT9*, *BCRP*, *MRP4*, nor *OAT10* mRNA expression was found in this study. In another study GLUT9 mRNA and protein expression was increased by uric acid [14]. In contrast with the previous study, N-acetylcysteine inhibited ROS production but did not affect GLUT9 expression. It is therefore uncertain how intracellular uric acid concentrations may have affected uric acid transporter expression in our experiments. Furthermore, a direct effect of febuxostat itself in our study cannot be ruled out.

Fourthly, whether NaDC3 plays a role in uric acid transport in HUVEC and HAEC is uncertain. This transporter is a driver of uric acid transport in the kidney where it transports dicarboxylates into the cell which are then exchanged for uric acid by other transporters [1]. However, the uric acid transporters in the panel we tested that utilize this gradient (URAT1, OAT1, OAT3, and OAT4) are not expressed in HUVEC and HAEC. Another study did report OAT10, which has been identified as another uric acid transporter by *in vitro* analyses and may utilize this gradient, to be present on HUVEC [13]. This transporter was not included in the panel we tested.

Fifthly, febuxostat increased mRNA expression of *GLUT9*, which functions as a uric acid importer in HUVEC [14], and decreased mRNA expression of *MRP4*, which functions as uric acid exporter in HUVEC [13]. In addition, febuxostat is a known inhibitor of BCRP [23]. The individual contribution of these effects on intracellular uric acid concentration is unknown. Our results indicate that apart from a direct modulation of BCRP protein function, febuxostat may also affect uric acid transporter expression by affecting gene transcription.

Sixthly, we did not investigate the downstream effects of the observed increase in intracellular uric acid concentration. Uric acid has been shown to induce ROS formation, nuclear factor-κB (NF-κB) activation, and NLRP3 inflammasome activation, among other pathways [12, 38]. The impact of varying intracellular uric acid levels on these pathways in our experiments remains unknown.

Finally, another limitation is that we only tested allopurinol but not oxypurinol. Oxypurinol is the active metabolite of allopurinol, has a longer half-life than allopurinol, and is responsible for most of the uric acid-lowering effect of allopurinol *in vivo* [31]. Oxypurinol can inhibit GLUT9-mediated uric acid uptake in Xenopus laevis oocytes [32]. Oxypurinol may therefore affect intracellular uric acid concentrations in HUVEC by reducing GLUT9-mediated uric acid uptake.

As mentioned previously, studies differ on which uric acid transporters are expressed in HUVEC. In accordance with our findings, three studies found mRNA expression of *GLUT9*, *BCRP*, and *MRP4*, but not *URAT1* [5, 13, 33]. Two of these studies additionally reported *MCT9* to be expressed [5, 33], while another found expression of *OAT10* [13]. Two other studies reported expression of URAT1 at the protein level [4, 34]. One of these studies also found expression of OAT1 and GLUT9 [4]. Komori et al. reported knockout of BCRP and MRP4 resulted in increased intracellular uric acid concentrations and ROS formation [13]. Liu et al. found that the glucose transport inhibitor phloretin significantly reduced GLUT9 expression, lowered uric acid uptake, and reduced inflammation markers in HUVEC [4]. Knockdown of GLUT9 resulted in a reduction in inflammation markers. Nie et al. detected that uric acid induced the expression of GLUT9, inflammation markers, and ROS formation [14]. GLUT9 knockdown resulted in reduced uric acid uptake and ROS formation. Taken together, these results show the importance of uric acid transporters in the regulation of intracellular uric acid concentration, uric acid induced inflammation, and oxidative stress in HUVEC.

Uric acid transporters may also play an important role in other cell types relevant for atherosclerosis. Martínez-Reyes et al. observed that human macrophages express URAT1 [35]. Uric acid induced the expression of inflammatory markers and increased phagocytic activity. Furthermore probenecid, a non-specific URAT1 inhibitor, reduced phagocytic and proinflammatory activity. The effects on intracellular uric acid accumulation and of XOI were not studied.

There are several opportunities for further research. The effects of uric acid, allopurinol, and febuxostat on the protein expression and localization of uric acid transporters should be explored further. Especially as Komori et al. reported a reduced BCRP plasma membrane expression while total protein and mRNA expression of BCRP remained constant [13]. The mechanism by which febuxostat affects the expression of uric acid transporters also requires further investigation. As oxypurinol can inhibit GLUT9-mediated uric acid uptake in Xenopus laevis oocytes [32], it is important to investigate whether oxypurinol can affect intracellular uric acid concentrations in HUVEC, especially as no effect of allopurinol was found in this study. Benzbromarone, a uric acid transporter inhibitor used in the treatment of gout, inhibits the activity of GLUT9, MRP4, and BCRP [22, 23, 36, 37], which raises the question how endothelial uric acid concentrations are affected by this drug. The impact of varying intracellular uric acid levels induced by allopurinol and febuxostat on inflammation, oxidative stress, and nitric oxide production should be investigated in HUVEC. This includes assessing effects on intracellular ROS concentration, the phosphoinositide 3-kinase (PI3K)/Akt pathway, the mitogen-activated protein kinase (MAPK) cascade, as well as NF-κB and NLRP3 inflammasome activation [12, 38]. These studies can provide valuable insights into the potential pathways through which febuxostat and allopurinol might impact the progression of atherosclerosis. Finally, the effects of febuxostat, allopurinol, and oxypurinol in other cell types relevant for the pathogenesis of atherosclerosis, such as macrophages, should be studied to better understand how these drugs may influence this disease.

## 5. Conclusion

Febuxostat significantly increased intracellular uric acid concentration in HUVEC compared with control, while allopurinol did not. We hypothesize that this difference in effect on endothelial uric acid handling explains the observed disparity in cardiovascular mortality between febuxostat and allopurinol in patients with gout.

## Supporting information

S1 Fig Intracellular uric acid concentrations in HUVEC treated with 350μM uric acid compared with 100μM uric acid. ***P<0.001; Box represents median, 25^th^, and 75^th^ percentile, whiskers represent range.
(TIF)

S2 Fig Cell count in HUVEC treated with 350μM uric acid compared with 100μM uric acid. Box represents median, 25^th^, and 75^th^ percentile, whiskers represent range; U350μM uric acid.
(TIF)
