# Supplementary figures and images for: Effects of allopurinol and febuxostat on uric acid transport and transporter expression in human umbilical vein endothelial cells
Source: PLoS One. 2024 Jun 21;19(6):e0305906. doi: 10.1371/journal.pone.0305906 (PMC11192402; doi:10.1371/journal.pone.0305906)

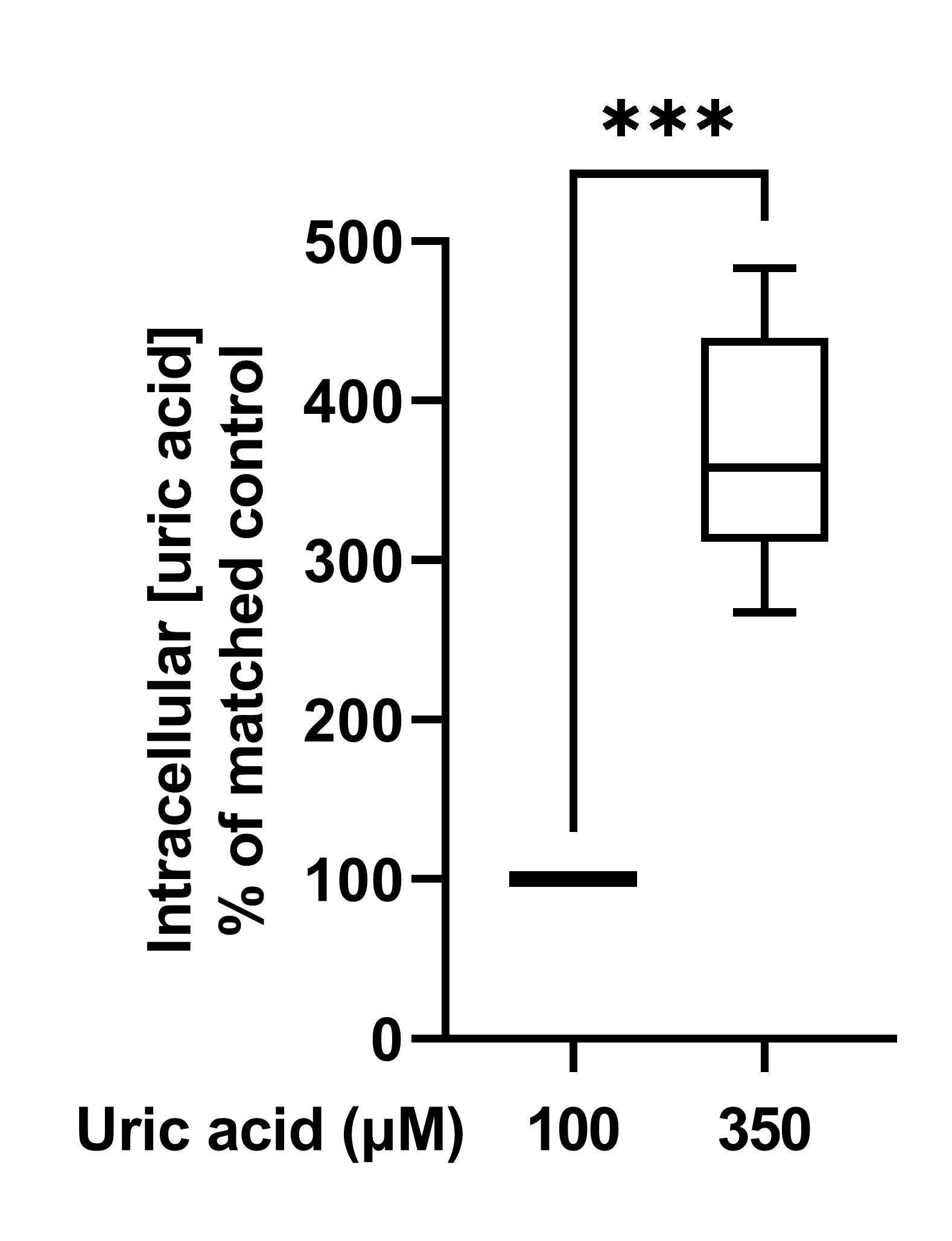

Supplement: S1 Fig — ***P<0.001; Box represents median, 25th, and 75th percentile, whiskers represent range. (TIF) [file pone.0305906.s001.tif]

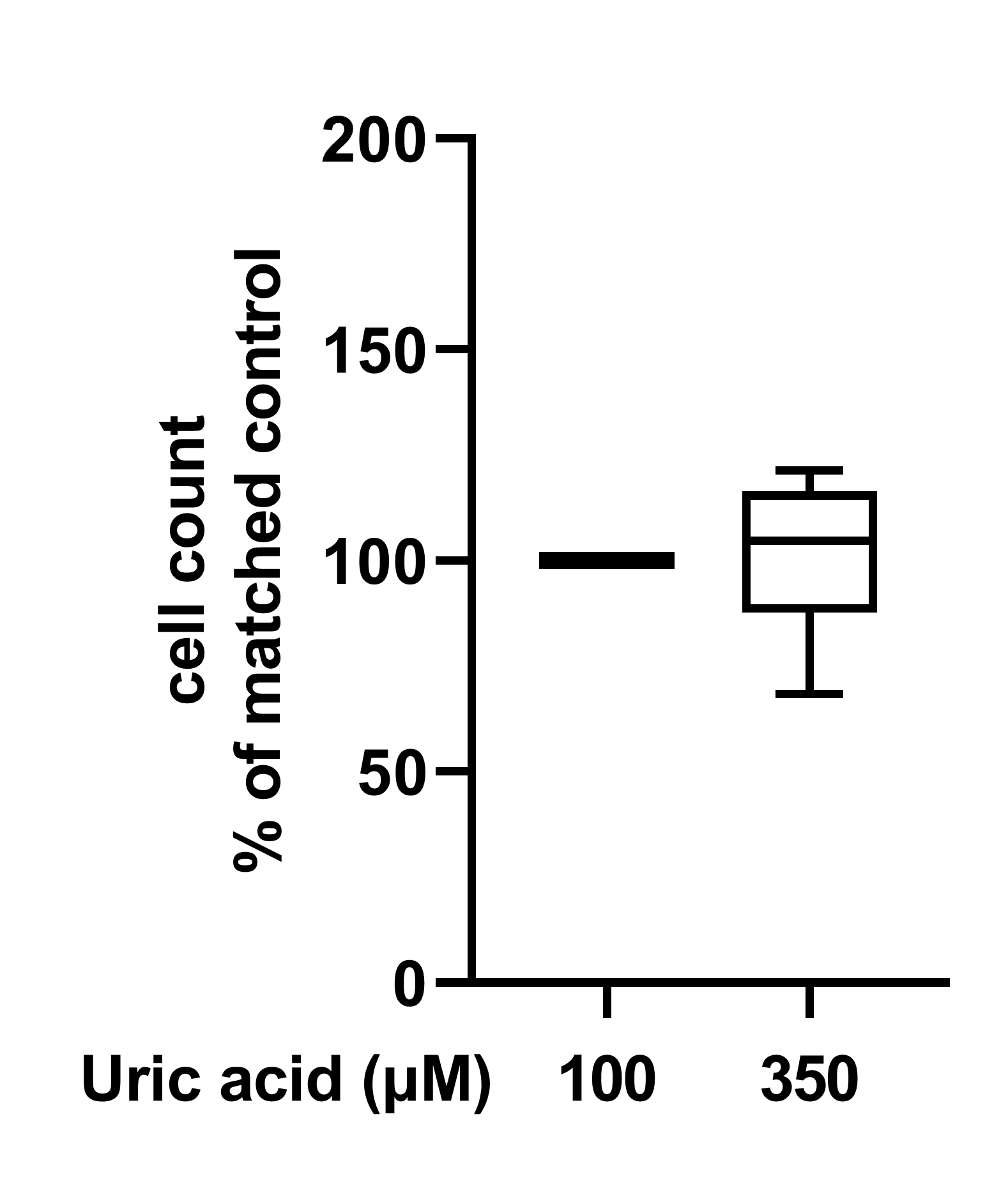

Supplement: S2 Fig — Box represents median, 25th, and 75th percentile, whiskers represent range; U350μM uric acid. (TIF) [file pone.0305906.s002.tif]
